# Enhanced physicochemical stability and efficacy of angiotensin I-converting enzyme (ACE) - inhibitory biopeptides by chitosan nanoparticles optimized using Box-Behnken design

**DOI:** 10.1038/s41598-018-28659-5

**Published:** 2018-07-10

**Authors:** Shehu Muhammad Auwal, Mohammad Zarei, Chin Ping Tan, Mahiran Basri, Nazamid Saari

**Affiliations:** 1 Department of Food Science, Faculty of Food Science and Technology, Universiti Putra Malaysia, 43400 Serdang, Selangor Malaysia; 2 Department of Food Technology, Faculty of Food Science and Technology, Universiti Putra Malaysia, 43400 Serdang, Selangor Malaysia; 3 Department of Chemistry, Faculty of Science, Universiti Putra Malaysia, 43400 Serdang, Selangor Malaysia; 4 Department of Biochemistry, Faculty of Basic Medical Sciences, Bayero University, Kano, Nigeria; 5 Department of Food Science and Technology, College of Agriculture and Natural Resources, Sanandaj Branch, Islamic Azad University, Sanandaj, Iran

## Abstract

Bromelain-generated biopeptides from stone fish protein exhibit strong inhibitory effect against ACE and can potentially serve as designer food (DF) with blood pressure lowering effect. Contextually, the DF refer to the biopeptides specifically produced to act as ACE-inhibitors other than their primary role in nutrition and can be used in the management of hypertension. However, the biopeptides are unstable under gastrointestinal tract (GIT) digestion and need to be stabilized for effective oral administration. In the present study, the stone fish biopeptides (SBs) were stabilized by their encapsulation in sodium tripolyphosphate (TPP) cross-linked chitosan nanoparticles produced by ionotropic gelation method. The nanoparticles formulation was then optimized via Box-Behnken experimental design to achieve smaller particle size (162.70 nm) and high encapsulation efficiency (75.36%) under the optimum condition of SBs:Chitosan mass ratio (0.35), homogenization speed (8000 rpm) and homogenization time (30 min). The SBs-loaded nanoparticles were characterized for morphology by transmission electron microscopy (TEM), physicochemical stability and efficacy. The nanoparticles were then lyophilized and analyzed using Fourier transform infra-red spectroscopy (FTIR) and X-ray diffraction (XRD). The results obtained indicated a sustained *in vitro* release and enhanced physicochemical stability of the SBs-loaded nanoparticles with smaller particle size and high encapsulation efficiency following long period of storage. Moreover, the efficacy study revealed improved inhibitory effect of the encapsulated SBs against ACE following simulated GIT digestion.

## Introduction

Marine invertebrates provide a unique source of multifunctional biopeptides with potential to exhibit wide array of physiological benefits on the body system^1^. The biopeptides are encrypted as inactive sequences within the primary structure of tissue protein and the biologically active forms can be released via properly designed enzymatic hydrolysis and under optimal condition of temperature, pH, E/S ratio and hydrolysis time for maximum yield^2,3^. The bioactive peptides have been associated with safe therapeutic effects and can be used as alternative to synthetic agents in the prevention and management of certain chronic diseases such as cardiovascular disease (CVD), diabetes, cancer and obesity-related chronic conditions^4,5^.

*Sea cucumber* is a protein-rich marine invertebrate. Numerous ACE-inhibitory biopeptides have been generated and characterized from different species of *sea cucumber* such as *Stichopus horrens*^6^; *Actinopyga lecanora* tissue proteins^7^; *Stichopus vastus* collagen^8^; *Isostichopus badionotus*^9^; *Acaudina malpadioidea* wall protein^10^. The ACE-inhibitory biopeptides derived from marine invertebrates were reported to exhibit *in vitro* inhibitory effect through both competitive^1,10^ and noncompetitive mechanisms^6^ on ACE. Additionally, *in vivo* studies in hypertensive rats have shown the ACE-inhibitory potential of marine-derived antihypertensive peptides^10,11^.

However, the bioactive peptides are susceptible to degradation by gastrointestinal enzymes and need to be protected for effective target site delivery via oral route. This can be achieved through encapsulation to safe guard their structural and functional integrity and improve their stability against gastrointestinal proteases and peptidases^12^. Hence, encapsulation has become a relevant and important technology to promote the utilization of bioactive peptides as functional food bioingredients for human health promotion. It protects the peptides against physicochemical modifications and enhances their efficacy both *in vitro* and *in vivo*^13,14^.

The selection of appropriate biocompatible and biodegradable wall material is necessary to meet safety requirements for health concern about their possible toxicity especially when nanocarriers are used for the delivery of the bioactive agents^15,16^. Chitosan is a cheap, natural, non-toxic and biodegradable polymer made up of (β1 → 4) linked 2-amino-2-deoxy-glucopyranose (GlcN) and 2-acetamido-2-deoxy-d-glucopyranose (GlcNAc) residues^17,18^. Consequently, it is considered as a safe coating material for the encapsulation of bioactive compounds^19,20^. The cationic amino residues of chitosan form complex via ionic gelation with oppositely charged non-toxic polyanionic molecules, like sodium tripolyphosphate^21^. The method is simple and doesn’t involve harsh treatment such as the use of organic solvents and high temperatures, hence applied for the encapsulation of fragile molecules, including peptides and proteins^22–26^.

The use of box behnken design (BBD) in process optimization is considered to be more effective and preferable compared to central composite design based on the less number of experiments that are involved in the former. To our knowledge, there is limited application of Box Behnken design to optimize the process condition for the fabrication of chitosan-entrapped biopeptides in the food industry. Consequently, the present work was aimed to improve the physicochemical stability and efficacy of bromelain-generated ACE-inhibitory biopeptides from stone fish as edible species of *sea cucumber* through their encapsulation in Chitosan:TPP nanoparticles using ionotropic gelation method and optimized by Box-Behnken design.

## Materials and Methods

### Chemicals and reagents

Low molecular weight (LMW) chitosan, sodium tripolyphosphate, angiotensin I-converting enzyme from rabbit lung and N-hippuryl-histidyl-leucine were purchased from Sigma Aldrich (St. Louis, MO, USA). Bromelain, Pepsin, Trypsin, Pancreatin, Tween 80, mono and dibasic potassium salts, sodium chloride, and sodium hydroxide were obtained from Merck (Darmstadt & Schuchardt, Germany). All other chemicals used were of analytical grade and purchased from Fisher scientific (Loughborough, UK), R & M (Essex, UK), Merck (Darmstadt, Germany) and Sigma Aldrich (St. Louis, MO, USA).

### Generation of stone fish biopeptides (SBs)

This was carried out according to Auwal *et al*.^2^. The stone fish powder was dialyzed against deionized water and then against phosphate buffer (50 mM, pH 7) in a 12–14 kDa molecular mass cut-off dialysis tube (MWCO). The dialyzed sample was preincubated at 40 °C and pH 7 in water bath shaker at 150 rpm followed by hydrolysis using bromelain at 2% E/S ratio for 240 min. The enzyme was then inactivated to stop the reaction by boiling the mixture for 10 min at 100 °C. The ACE-inhibitory biopeptides with mw <10 kDa present in the supernatant were separated and concentrated by centrifugation at 10,000 rpm and 4 °C for 20 min using Vivaspin® 20 ultrafiltration membrane device (MWCO 10 kDa, Sartorius). This was then filtered through a 0.45 µm non-protein binding membrane, freeze dried and stored at −40 °C before analysis. The biopeptides content was determined as serine amino equivalent from a calibrated curve constructed using L-serine.

### Experimental design and model building

Response surface methodology based on Box–Behnken design was used for building second order polynomial models to optimize the Chitosan:TPP nanoparticles formulation using MINITAB software Version 16.0. Three factors were employed at three levels to minimize particle size and maximize encapsulation efficiency by evaluation of the main, interaction, and quadratic effects of SBs:Chitosan mass ratio, homogenization speed (rpm) and homogenization time (min) on particle size and encapsulation efficiency.

The Box–Behnken design comprises of 15 experimental runs with three centre points and was selected in preference to central composite design because less number of runs is involved. The nonlinear equation for the quadratic model or its reduced form is given as;

1 $${\rm{R}}={{\rm{\beta }}}_{0}+{{\rm{\beta }}}_{1}{\rm{A}}+{{\rm{\beta }}}_{2}{\rm{B}}+{{\rm{\beta }}}_{3}{\rm{C}}+{{\rm{\beta }}}_{12}{\rm{AB}}+{{\rm{\beta }}}_{13}{\rm{AC}}+{{\rm{\beta }}}_{23}{\rm{BC}}+{{\rm{\beta }}}_{11}{{\rm{A}}}^{2}+{{\rm{\beta }}}_{22}{{\rm{B}}}^{2}+{{\rm{\beta }}}_{33}{{\rm{C}}}^{2}$$

where R is response, β_0_ is intercept and β_1_, β_2_, β_3_, β_11_, β_12_, β_13_, β_22_, β_23_ and β_33_ are regression coefficients calculated from the values of R obtained from the experiments. A β-coefficient with positive sign indicates synergistic effect while a β-coefficient with negative sign indicate the antagonistic effect of the process variables; A (SBs:Chitosan mass ratio), B (Homogenization speed, rpm) and C (Homogenization time, min) respectively, AB, AC and BC are interaction terms while A^2^, B^2^ and C^2^ are the quadratic terms of the models. The low, medium and high levels of the process variables were selected based on a preliminary experiment to produce the nanoparticles formulation with the most desirable properties.

### Statistical optimization, data analysis, and model validation

The response factors were fitted to linear, second order, and quadratic models for statistical validation of the polynomial equation. The statistical significance of β-coefficients and coefficient of determination (R-squared) values were used to evaluate the fitness of the models and the significant effects of the process variables on the responses using analysis of variance (ANOVA) according to Minitab 16.0 software. Results are expressed as average of replicate values ± standard deviation at a significance level of p < 0.05.

**Figure 1:**
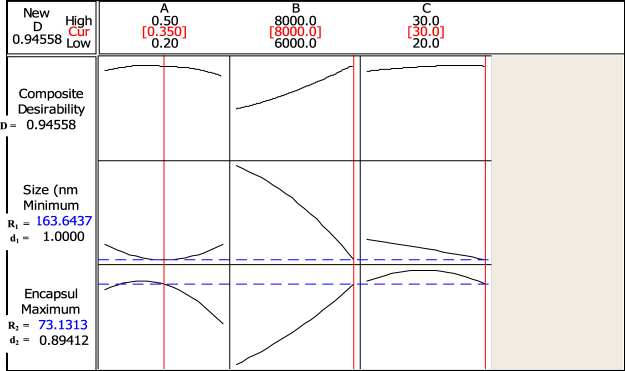
Response optimization for the formulation of SBs loaded Chitosan:TPP NPs, composite and individual desirabilities (D, d_1_ and d_2_) of the predicted outcomes R_1_ = particle size (nm), R_2_ = Encapsulation efficiency (%), A; (SBs:Chitosan mass ratio), B; (Homogenization speed and C; (Homogenization time).

**Figure 2:**
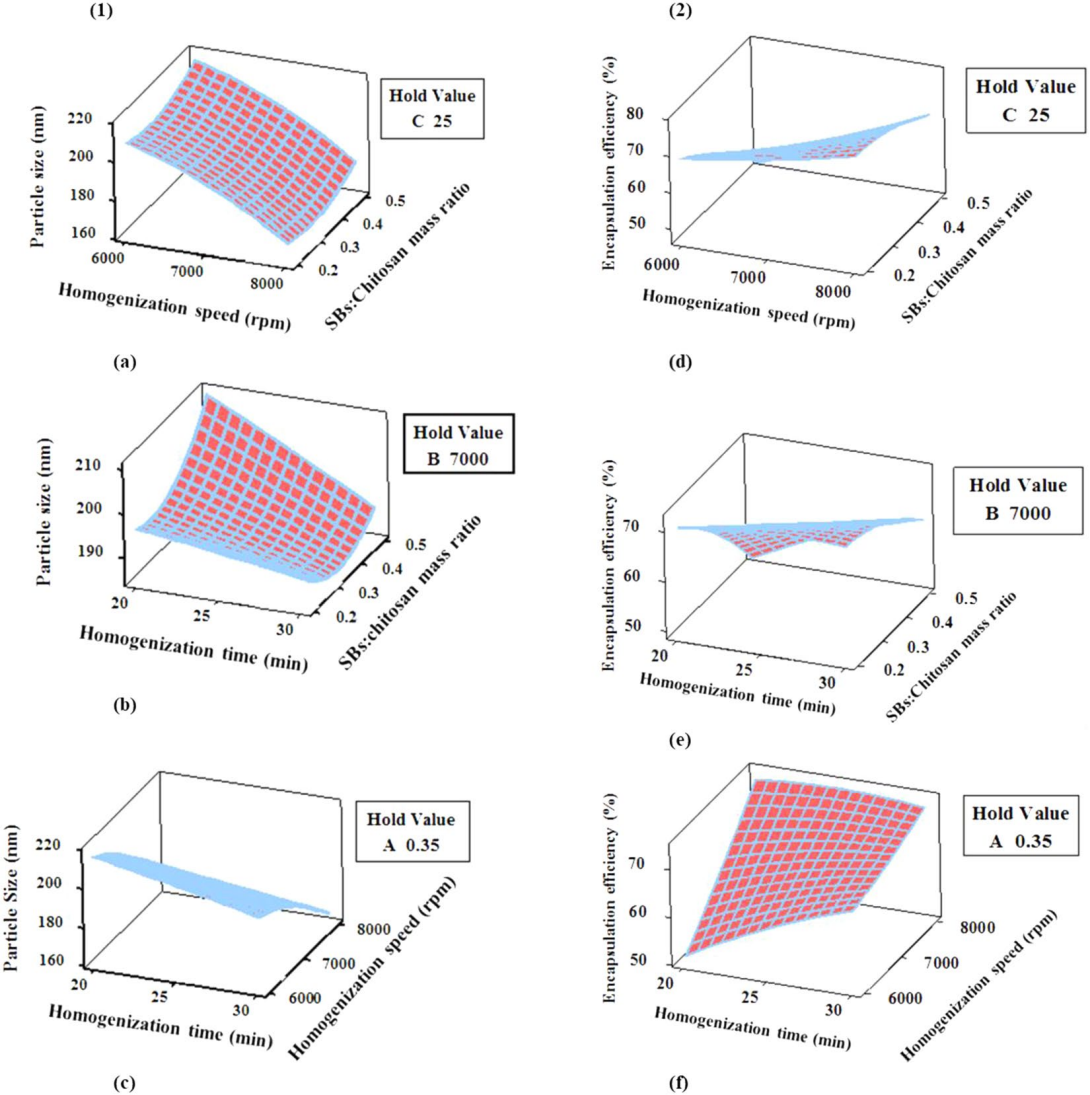
Three dimensional response surface plots for the effect of process conditions on; (1). Particle size (nm): (**a**) SBs:Chitosan mass ratio (A) and Homogenization speed (B); (**b**) SBs:Chitosan mass ratio (A) and Homogenization time (C); (**c**) Homogenization speed (B) and Homogenization time (C), (2). Encapsulation efficiency (%): (**d**) SBs:Chitosan mass ratio (A) and Homogenization speed (B); (**e**) SBs:Chitosan mass ratio (A) and Homogenization time (C); (**f**) Homogenization speed (B) and Homogenization time (C).

The theoretical optimum levels of the independent factors to produce nanoparticles formulation with small size and high encapsulation efficiency were determined using response optimizer (Fig. 1). The response optimizer is an integral component of optimisation using response surface methodology. It predicts the best levels of the independent variables (encapsulation conditions) that will jointly yield optimum responses (small particle size and high encapsulation) for statistically fitted models. Experiments were carried out under the same encapsulation conditions for statistical validation of the theoretical/predicted responses. The relationship between the responses and the process conditions are illustrated by response surface plots (Fig. 2).

The fitness of the final models was further ascertained by comparing the response values with the fitted values predicted by response regression equation^2,27^.

### Preparation of stone fish biopeptides loaded Chitosan:TPP nanoparticles (SBs loaded Chitosan:TPP NPs)

The SBs loaded Chitosan:TPP NPs were produced by ionotropic gelation method as previously described^28^. Chitosan solution was prepared at 0.5%w/v (5 mg/mL) in 1% acetic acid and added to a mixture of SBs (mw <10 kDa) and Tween 80 prepared at a concentration of 5 mg/mL and 0.5% (v/v) in deionized water, respectively. The mixture was sheared at 25 °C using high shear homogenizer (IKA® T 25 digital ULTRA-TURRAX®) according to the conditions designed for each experiment (Table 1). The TPP prepared at 0.5% (w/v) was then added drop-wise to the stirring solution. Different SBs:Chitosan mass ratios were used (Table 1) at a fixed mass ratio of 4:1 for Chitosan:TPP determined from preliminary screening of the formulation parameters. The resulting nanoparticle suspension was sonicated for 10 min in an ice bath and adjusted to pH 5.5 before further analysis.

**Table 1:** Experimental and predicted values of response variables for Box-behnken design.

Run order	^a^Independent variables			^b^Dependent variables			
	A	B	C	R_1_ (Mean particle size, nm)		R_2_ (Encapsulation efficiency, %)	
				Predicted	Experimental	Predicted	Experimenta
1	0.35	8000	30	163.755	164.90	73.3484	73.92
2	0.50	7000	20	210.328	210.78	50.4725	49.93
3	0.35	6000	20	215.238	213.87	51.7059	50.70
4	0.20	6000	25	208.456	209.63	67.8563	69.23
5	0.20	7000	30	190.645	189.97	72.6800	71.66
6	0.20	8000	25	169.409	168.97	75.8688	76.10
7	0.20	7000	20	196.000	195.94	71.0450	70.46
8	0.35	7000	25	191.362	190.00	66.3271	65.61
9	0.50	6000	25	215.704	216.65	46.7288	48.06
10	0.35	8000	20	171.140	171.67	73.5934	73.73
11	0.35	7000	25	191.362	190.94	66.3271	65.62
12	0.35	7000	25	191.362	193.59	66.3271	68.62
13	0.50	8000	25	176.656	175.42	67.2912	67.48
14	0.35	6000	30	197.753	197.00	66.6609	66.09
15	0.50	7000	30	190.813	190.65	63.5475	62.57

^a^Independent variables for encapsulation of SBs: A; SBs:Chitosan mass ratio, B; homogenization speed (rmp), C; homogenization time (min).

^b^Dependent variables: R_1_; mean particle size (nm); R_2_; encapsulation efficiency (%).

### Evaluation of encapsulation efficiency of the SBs loaded Chitosan:TPP NPs

The encapsulation efficiency of the SBs loaded Chitosan:TPP NPs was determined as the percentage ratio of the encapsulated biopeptides to the total biopeptides according to the equation;

2 $${\rm{EE}}( \% )={\rm{Ebp}}/{\rm{Tbp}}\times {\rm{100}}$$

The amount of the encapsulated biopeptides (Ebp) was indirectly determined as the difference between the total amount of biopeptides initially added (Tbp) and that of the unencapsulated biopeptides (UEbp) given as;

3 $${\rm{Ebp}}={\rm{Tbp}}-{\rm{UEbp}}.$$

The UEbp was separated by centrifugation of the nanoparticles suspension at 10,000 rpm and 4 °C for 20 min. The free biopeptides content was then quantified by modified Lowry method using Bicinchoninic Acid (BCA) micro protein Assay kit (Sigma-Aldrich, USA).

### Determination of particle size, pdi and zeta potential (ζ-potential) of the SBs loaded Chitosan:TPP NPs

The mean diameter, polydispersity index and ζ-potential of the SBs loaded Chitosan:TPP NPs were determined by dynamic light scattering using a particle size analyzer (Malvern, Zetasizer Nano Series, UK). The nanoparticles suspension was appropriately diluted and transferred into disposable capillary tube. The experiment was carried out at 25 °C and measurements were taken in five replicates.

### *In vitro* release of the SBs loaded Chitosan:TPP NPs

The *in vitro* release of SBs loaded Chitosan:TPP NPs was evaluated by dialysis method^29^. A 12–14 kDa MWCO dialysis bag containing 20 mL of the nanoparticles suspension was suspended in 200 mL phosphate buffer pH 7.4 as the release medium to which 1% Tween 80 (v/v) was added to maintain a sink condition. The experiment was carried out in a water bath at 250 rpm and 37 °C for 12 h. At a specific time interval, a 1 mL of the nanoparticles suspension was withdrawn and used to determine change in encapsulation efficiency of the nanocapsules as follows;

4 $${\rm{Release}}\,{\rm{ratio}}( \% )={\rm{1}}-({{\rm{EE}}}_{{\rm{t}}}/{{\rm{EE}}}_{0})\times {\rm{100}}$$

where EE_0_ is the encapsulation efficiency before dialysis, EE_t_ is the encapsulation efficiency for the sample taken at a time “t” during dialysis.

### *In vitro* digestion and ACE- inhibitory activity of the SBs loaded Chitosan:TPP NPs under simulated-gastric-fluid (SGF) and simulated-intestinal-fluid (SIF)

The *in vitro* digestion of the SBs loaded Chitosan:TPP NPs was evaluated as previously described^30–32^ and ACE-inhibitory activity was determined by colorimetric assay^33^. The SBs loaded Chitosan:TPP NPs were incubated in simulated-gastric-fluid (SGF), and then in simulated-intestinal-fluid (SIF). The SGF was made by mixing 20 mg/mL of pepsin, 350 µL of concentrated HCl and 0.1 g of NaCl in a total volume of 50 mL with deionized water and adjusted the pH to 1.2. A 1 mL solution of the SGF was added to 3 mL of the nanoparticles suspension and incubated at 37 °C under magnetic stirring for 60 min.

The SIF solution was made by mixing 34 mg/mL of monobasic potassium phosphate, 3.85 mL of 200 mM NaOH and 0.5 g of pancreatin to a final volume of 50 mL with deionized water and adjusted the pH to 6.8 with 0.5 M NaOH.

Then, 1 mL of the SIF solution was added to the reaction mixture and re-incubated for another 60 min under the same condition. The suspension containing the digested product was boiled at 100 °C for 10 min to inactivate enzymes. The suspension was then cooled to room temperature and adjusted the pH to 5.5. This was then stirred to completely disrupt the residual nanoparticles to release their SBs contents and the ACE-inhibitory activity was determined after centrifugation.

### Physicochemical Stability Studies

The Chitosan:TPP nanoparticles loaded with the ACE-inhibitory SBs were stored in universal bottles at 4 °C and evaluated for physicochemical stability by monitoring changes in particle size, pdi, zeta potential and encapsulation efficiency during the 12 weeks storage period.

### Fourier transform infrared spectroscopy (FTIR)

The Fourier transform infrared (FTIR) spectra of the freeze dried Empty Chitosan:TPP NPs and SBs loaded Chitosan:TPP NPs were obtained as a mean of sixteenth scans collected at a resolution of 4 cm^−1^ using an ATR - FTIR spectrometer (Spectrum 100 PerkinElmer), in the region of 4000–400 cm^−1^. The differences in spectral peaks of the Empty Chitosan:TPP NPs and SBs loaded Chitosan:TPP NPs were then evaluated.

### X-ray diffraction (XRD) analysis

Lyophilized nanoparticles were analyzed using an X-ray diffractometer (Phillips X′PERT, copper anode, λ = 1.5418 angstroms), and the data were obtained from scans in the range of 2θ from 5 to 80° (steps of 0.0330°), at 25 °C.

### Transmission electron microscopy (TEM)

The morphology of the SBs loaded Chitosan:TPP NPs was determined using transmission electron microscopy (TEM) (Hitachi H-7100 TEM, Hitachi Ltd., Chiyoda, Japan). The nanoparticles were applied onto a copper grid and air dried for 3 min at room temperature. The nanoparticles loaded grid was then negatively stained using 1% uranyl acetate for 90 s and air dried at room temperature before the transmission electron microscopic examination.

### Data availability

The data obtained from the study are with the corresponding author and can be made available on request.

## Results and Discussion

### Graphical optimization and Model fitting

The SBs loaded Chitosan:TPP NPs were produced by ionotropic gelation method under different encapsulation conditions according to response surface methodology by Box-Behnken design (Table 1). The main effect, interaction effect and quadratic effect of the three independent variables including; SBs:Chitosan mass ratio (A), homogenization speed (B) and homogenization time (C) were analyzed on the particle size (R_1_) and encapsulation efficiency (R_2_) as the response variables of the resulting nanoparticles (Table 1).

The graphical optimization which determine the best combination of the independent variables to produce SBs loaded Chitosan:TPP NPs with lower particle size and higher EE was carried out using response optimizer (Fig. 1).

The predicted optimum levels of the three independent variables including A (0.35), B (8000 rpm) and C (30 min) were used to generate optimized SBs loaded Chitosan:TPP NPs formulations with particle size (R_1_) and EE (R_2_) of 162.70 nm and 75.36%, respectively. No statistical difference was observed compared to the corresponding predicted values of 163.64 nm and 73.13% for R_1_ and R_2_, respectively. The desired responses under the optimum conditions showed a composite desirability of 0.95 with individual desirabilities of 1 and 0.89 for R_1_ and R_2_, respectively.

The results for the ANOVA of the fitted quadratic polynomial models are given in Table 2.

**Table 2:** Analysis of variance for mean particle size (R_1_) and Encapsulation efficiency (R_2_) of the fitted quadratic models.

Source	SS		DF		MS		F		P		Remarks
	R_1_	R_2_	R_1_	R_2_	R_1_	R_2_	R_1_	R_2_	R_1_	R_2_	
Model	3740.83	1100.85	7	7	534.404	157.265	238.11	76.79	0.000	0.000	Significant
A	1.83	49.70	1	1	1.835	49.698	0.82	24.27	0.396	0.002	
B	14.53	45.80	1	1	14.529	45.798	6.47	22.36	0.038	0.002	
C	17.79	40.56	1	1	17.791	40.561	7.93	19.81	0.026	0.003	
A*A	115.85	13.35	1	1	115.849	13.348	51.62	6.52	0.000	0.038	
B*B	71.59		1		71.588		31.90		0.001		
C*C											
A*B		39.38		1		39.376		19.23		0.003	
A*C	50.13	32.72	1	1	50.126	32.718	22.33	15.98	0.002	0.005	
B*C	25.50	57.76	1	1	25.503	57.760	11.36	28.20	0.012	0.001	
Residual Error	15.71	14.34	7	7	2.244	2.048					
Pure Error	6.93	6.02	2	5	3.466	3.010					
Lack-of-Fit	8.78	8.32	5	2	1.756	1.663	0.51	0.55	0.767	0.744	Non-Significant
Total	312.93	307.95	14	14							
R_1_ (R^2^ = 99.58%, R^2^ – pred. = 98.02%, R^2^ – adj. = 99.16%, S.D = 1.50, PRESS = 74.51											
R_2_ (R^2^ = 98.71%, R^2^ – pred. = 93.98%, R^2^ – adj. = 97.43%, S.D = 1.43, PRESS = 67.11											

The terms of the independent factors with non-significant effects (P > 0.05) were eliminated from the models and the two response factors were fitted to linear, interaction, and quadratic models for statistical validation of the polynomial equation.

The fitted polynomial equations for the effect of the independent factors on the particle size (R_1_) and %EE (R_2_) are given as follows;

5 $${{\rm{R}}}_{1}=213.02\,-\,31.59{\rm{A}}+0.029{\rm{B}}\,-\,3.13{\rm{C}}\,-\,4.72{\rm{AC}}+0.001{\rm{BC}}+248.21{{\rm{A}}}^{2}$$

6 $${{\rm{R}}}_{2}=-\,43.42\,-\,232.43{\rm{A}}+0.019{\rm{B}}+4.72{\rm{C}}+0.021{\rm{AB}}+3.81{\rm{AC}}\,-\,0.001{\rm{BC}}\,-\,84.04{{\rm{A}}}^{2}$$

The factors B, C, A^2^ and the interactions AC and BC were significant for particle size modelling while the variables A, B, C, A^2^ and interactions terms AB, AC and BC were significant in the model developed for %EE.

The modelling in equations (5) and (6) above showed that the particle size (R_1_) and encapsulation efficiency (R_2_) were dependent on C and increase in this factor lead to decreased particle size and increased EE. Increase in factor A due to higher load of SBs and low chitosan mass, decreased both R_1_ and R_2_. However, increase in factor B resulted in increased R_1_ and higher R_2_ at low SBs load and high chitosan mass ratio. Similarly, the increase in the encapsulation efficiency of chitosan nanoparticles was associated with low release due to decreased protein loading and increased chitosan concentration^34^. This could be related to the high potency of the chitosan to form ionic gel that prevented the leakage of the biopeptides to the external phase and enhanced their entrapment efficiency. However, the observed increase in particle size and high EE due to increased homogenization speed is not in line with the previous finding^35^ and could be due to higher chitosan mass with more available surfaces to ionically gelate the crosslinking TPP and entrapped more SBs at low loading. In addition, prolonged homogenization time and low chitosan concentration were associated with leakage of the bioactive compound and decreased EE^36^.

The interaction and quadratic terms of regression equation involved more than one factor and factor of higher order respectively, and suggest non-linear relationship between the independent factors and their responses^37^. The three dimensional (3D) response surface plots for the interaction effects due to two pair of factors (AB, AC and BC) on particle size (R_1_) and encapsulation efficiency (R_2_) are shown in Fig. 2. Figure 2a,d illustrated the combined effect of SBs:Chitosan mass ratio (A) and homogenization speed (B) on the R_1_ and R_2_ responses at fixed homogenization time (C) of 25 min. The B had a synergistic effect on both R_1_ and R_2_ while A showed an antagonistic effect that was not significant on R_1_ but significant on R_2_ at p < 0.05 (Table 2). The interaction term AB does not show significant effect on R_1_ and was deleted from the model. However, the interaction of AB yielded positive effect on R_2_. The 3D plots in Fig. 2b,e displayed the combined effect of A and C on R_1_ and R_2_ by holding B at 7000 rpm. The C showed an antagonistic effect on R_1_ and a synergistic effect on R_2_ that were both significant at p < 0.05. However, A exhibited antagonistic effect that was only significant on R_2_. The interaction between A and C resulted in their significant negative effect on R_1_ and positive effect on R_2_. The plots in Fig. 2c,f demonstrated combined effect of B and C on R_1_ and R_2_ at a fixed value of 0.35 for A. The B indicated significant synergistic effect on both R_1_ and R_2_ while C showed antagonistic effect on R_1_ and synergistic effect on R_2_ and were both significant at p < 0.05. Moreover, interaction between B and C revealed a significant synergistic effect on R_1_ and antagonistic effect on R_2_. Only A^2^ was found to have significant quadratic effect on R_1_ and R_2_. The term, A^2^ have positive effect and high magnitude on R_1_ but affected R_2_ negatively.

The full quadratic models showed the highest R^2^ values of 99.58% and 98.71% for R_1_ and R_2_ responses and were selected as the best fit models (Table 2). Accordingly, the models *F*-values of 238.11 and 76.79 revealed their great significance on the R_1_ and R_2_ responses. The significance of each quadratic model is further proven by the *F*-value probability of p < 0.05 obtained for both R_1_ and R_2_. In addition, the insignificant lack of fit values for R_1_ (0.767) and R_2_ (0.744) indicated strong correlation between the independent and dependent variables of the models. The slight difference observed in the values of R^2^-adjusted and R^2^-predicted for both R_1_ and R_2_ indicated the validity and statistical significance of the equations in the optimization of the SBs loaded Chitosan:TPP NPs. In addition, the small values of the predicted residual sum of squares (PRESS) for R_1_ and R_2_ indicated better fit of the models to the data points in the design^38^.

### Physicochemical stability

**Figure 3:**
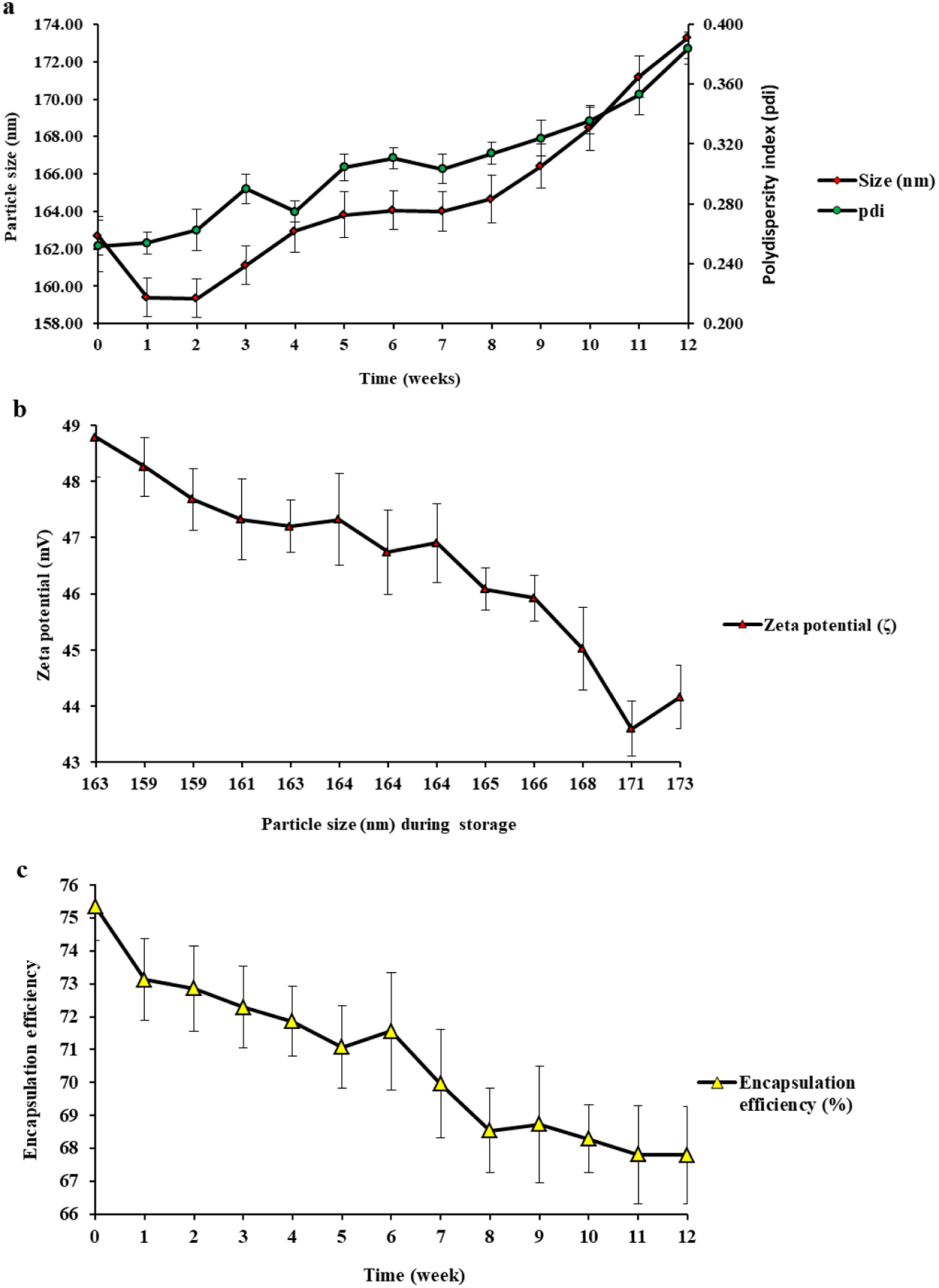
Changes in the physicochemical properties of the optimized Chitosan:TPP NPs containing SBs during 12 weeks of storage at 4 °C (**a**) Particle size and polydispersity index (pdi) (**b**) zeta potential (ζ) and (**c**) Encapsulation efficiency.

The optimized SBs loaded Chitosan:TPP nanoparticles were stored at 4 °C and evaluated for physicochemical stability once weekly for a period of 12 weeks. The physical stability was assessed by monitoring the variation in size, pdi and zeta potential while the chemical stability was determined by measuring the changes in encapsulation efficiency. A parallel change in particle size and pdi was observed. The mean particle size and pdi of the freshly prepared nanoparticles were 162.70 nm and 0.252, respectively. After the 12 weeks of storage, the particle size and pdi were 173.30 nm and 0.384, respectively (Fig. 3a). The zeta potential was found to decrease from +48.78 to +43.60 (Fig. 3b) while encapsulation efficiency was between 75.36% to 67.79% (Fig. 3c).

There were no significant changes in the mean particle size up to the eight week and in pdi up to twelve week of storage at 4 °C as evaluated by ANOVA at p < 0.05 using Fisher’s significance test. The observed significant changes in particle size (P < 0.05) after the eight week, might be attributed to the swelling of the nanoparticles due to their characteristic water absorption ability. Whereas the significant decrease in encapsulation efficiency (p < 0.05) might have occurred due to the release of SBs from the nanocapsules resulting from the swelling and degradation of the SBs loaded Chitosan:TPP NPs in the aqueous medium due to long term period of storage^36,39^. However, no significant change was seen in zeta potential (p < 0.05) during the first ten weeks of storage. This was demonstrated by the high positive zeta potential maintained which revealed strong electrostatic repulsion between the nanoparticles that prevented their aggregation and increased their colloidal stability.

The non-significant increase in pdi (<0.5) indicated little or no aggregation and higher dispersion of the nanoparticles suspension. Thus, the non-lyophilized suspension of the SBs loaded Chitosan:TPP NPs prepared by ionotropic gelation demonstrated high stability during the long storage time. This showed their suitability as effective carrier systems for successful target site delivery of the encapsulated bioactive peptides.

### FTIR spectroscopy

**Figure 4:**
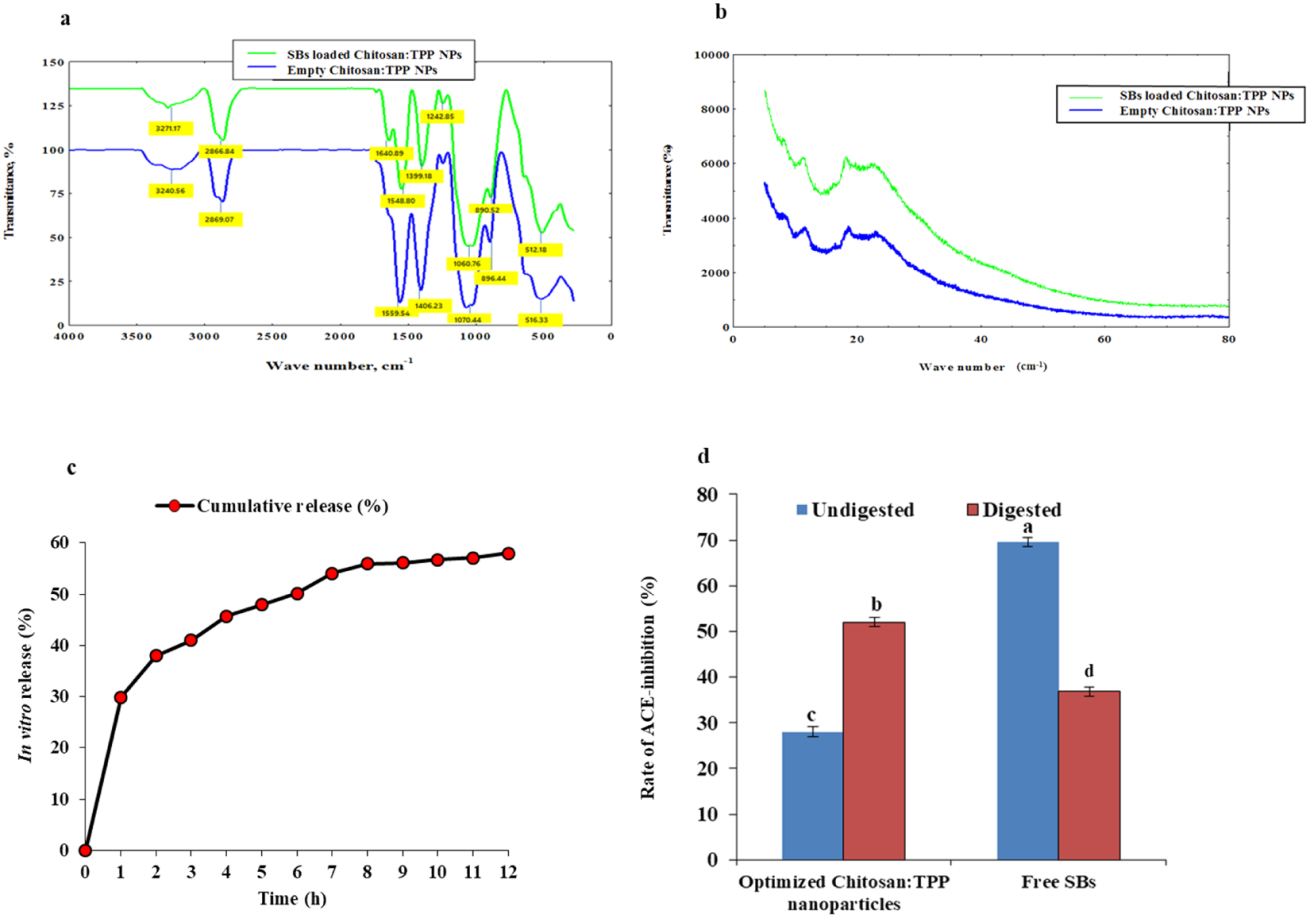
Physicochemical characterization of Empty Chitosan:TPP NPs and SBs loaded Chitosan:TPP NPs: (**a**) FTIR spectra of the Empty Chitosan:TPP NPs and SBs loaded Chitosan:TPP NPs; (**b**) X ray diffractogram of the Empty Chitosan:TPP NPs and SBs loaded Chitosan:TPP NPs; (**c**) *In vitro* cumulative release profile of the encapsulated SBs from the SBs loaded Chitosan:TPP NPs following 12 h of incubation in phosphate buffered saline, pH 7.4; (**d**) ACE-inhibitory activity of free SBs and SBs loaded Chitosan:TPP NPs before and after simulated GIT digestion, different letters indicate significant difference (p <0.05).

The spectral peaks of the Empty Chitosan:TPP NPs and SBs loaded Chitosan:TPP NPs were used to elucidate the distribution and nature of interaction of the SBs within the chitosan nanocapsules. The wave numbers of the FTIR absorption spectra corresponding to the functional groups that are involved in the interaction between the SBs and the chitosan matrix are shown in Fig. 4a. The wide shift in the spectral peak of Empty Chitosan:TPP NPs from 3240.56 cm^−1^ to 3271.17 cm^−1^ is related to (O-H) stretch and indicated the enhancement of the OH group in the SBs loaded Chitosan:TPP NPs. The carbonylated compounds stretch (C-H) of the polar surface of the Empty Chitosan:TPP NPs at 2869.07 cm^−1^ was not influenced with the loaded SBs suggesting low or no interaction of the SBs with the surfaces of the surrounding chitosan. The shift in the wave number of the empty nanocapsules from 1559.54 cm^−1^ to 1548.80 cm^−1^ in the SBs loaded nanoparticle plus the presence of an additional peak at 1640.89 cm^−1^ is typical of the vibrational bending stretch of N-H of amine І and amide ІІ carbonyl stretch and might be due to the amino acids content of the encapsulated biopeptides and indicated the distribution of the SBs and their interaction with the inner matrix of the chitosan nanocapsules. The inclusion of the SBs shifted the spectral peaks representing C-O-H bending from 1406.23 cm^−1^ in the empty capsule to 1399.18 cm^−1^ which suggested more interaction of the SBs with the chitosan matrix. The slight shift in the C-O stretch from 1243.54 cm^−1^ to 1242.85 cm^−1^ due to SBs load demonstrated little interaction with their coating surfaces of chitosan. Moreover, the loaded SBs resulted in the shift of asymmetric stretch of C-O-C from 1070.44 cm^–1^ to 1060.76 cm^−1^, C-H and CH_2_ bending from 896.44 cm^−1^ to 890.52 cm^−1^ which showed more interactions in the SBs loaded Chitosan:TPP NPs. The vibrational shift in the wave number from 1070.44 cm^–1^ to 1060.76 cm^−1^ might also be attributed to the linkage between phosphoric residues of TPP and ammonium ion in the chitosan. Thus, the net effects of the interactions between the chitosan and the TPP cross linkages and between the SBs and their surrounding Chitosan-TPP matrix contributed to the stability of the SBs loaded Chitosan NPs.

### XRD Analysis

The X-ray diffraction pattern showed that both the Empty Chitosan:TPP NPs and SBs loaded chitosan:TPP NPs were completely amorphous (Fig. 4b). The few and broaden peaks observed in the XRD pattern of SBs loaded Chitosan:TPP NPs indicated their amorphous nature or non-crystallinity^40^ and can be attributed to the cross linking interactions between chitosan and TPP around the SBs. This confirmed the amorphous nature of SBs and suggested their entrapment by finely coated cross linkages of chitosan-TPP. Similarly, the absence of sharp peak in the XRD diffractogram of nanoparticles was related to the complexation among counter ions which enable more intermolecular interaction between bioactive compound and the surrounding chitosan matrix, ascertaining the successful incorporation of the SBs into the core of the nanocapsules. This also supported the results obtained for FTIR and TEM. The amorphous nature is a desirable property for the SBs loaded Chitosan:TPP NPs which means they are more soluble or much easier to rehydrate compared to the crystalline assembly^41^.

### *In vitro* SBs release

The entrapment of SBs within the TPP cross-linked chitosan nanoparticles resulted in their prolonged retention and sustained release which indicated the improved bioavailability and long term therapeutic effect of the biopeptides at the target site. As shown in Fig. 4c, the SBs were released from the nanoparticles suspension within the 12 h of incubation in phosphate buffered saline, pH 7.4. The release of SBs from the chitosan nanoparticles involved a biphasic mode with an initial burst of rapid release at 29.87% during the first hour followed by a period of slow release at an almost constant rate to achieve a controlled release profile with cumulative SBs release of 58.0%. This is in agreement with the finding on the Curcumin loaded chitosan nanoparticles^42^. The burst release effect observed during the initial phase might be due to the desorption and diffusion of the SBs that were loosely adsorbed or associated to the surface of chitosan nanoparticles whereas the slow release phase might be attributed to the slow degradation of the nanoparticles. Thus, the release mechanism of the encapsulated SBs involves the degradation of the nanoparticles and the diffusion of the biopeptides across their surrounding layer of crosslinked Chitosan:TPP into the suspending medium.

### *In vitro* gastrointestinal digestion and ACE-inhibitory activity

The ACE-inhibitory activity of undigested and digested free SBs, and SBs loaded Chitosan:TPP NPs are shown in Fig. 4d.

It can be seen that the free undigested SBs and digested SBs loaded Chitosan:TPP NPs showed significantly higher (p < 0.05) ACE-inhibitory activity of 69.56% per 0.118 mg solid biopeptides and 51.96% per 0.085 mg solid biopeptides compared to their corresponding digested free SBs and undigested SBs loaded Chitosan:TPP NPs at 36.84% per 0.067 mg solid biopeptides and 28.06% per 0.025 mg solid biopeptides, respectively. Thus, the digested SBs loaded Chitosan:TPP NPs demonstrated greater efficacy with residual ACE-inhibitory activity of 51.96% at 0.085 mg solid biopeptides that was significantly higher (p < 0.05) than 36.84% obtained for the digested free SBs at 0.067 mg solid biopeptides. It has been reported that simulated gastrointestinal digestion resulted in decreased ACE-inhibitory activity of some peptides. However, some peptides were reported to resist gastrointestinal digestion and in some cases lead to shorter peptides with more potent inhibitory activity against ACE^43^. The decreased ACE-inhibitory activity observed for the free SBs is an indication that the biopeptides exhibited low resistant against digestive enzymes.

### Transmission electron microscopy (TEM)

**Figure 5:**
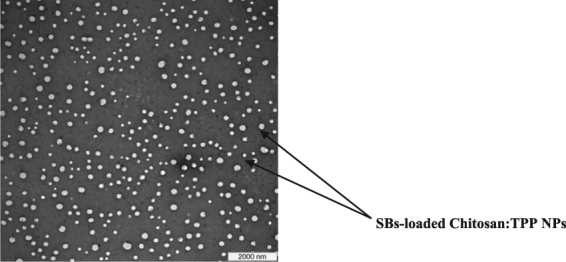
TEM images of the SBs loaded Chitosan:TPP NPs.

As shown in Fig. 5, the TEM images indicated that the SBs loaded Chitosan:TPP NPs are discrete and homogenously dispersed. This observation is further supported by the low pdi values (<0.5) of the nanoparticles suspension.

## Conclusion

In the present study, SBs loaded Chitosan:TPP NPs were prepared by ionotropic gelation method and optimized by a 3 factor-3 level-Box Behnken design. The process conditions including SBs:Chitosan mass ratio (0.35), homogenization speed (8000 rpm) and homogenization time (30 min) were successfully established as the optimum for the fabrication of the nanoparticles with minimum particle size (162.70 nm) and maximum encapsulation efficiency (75.36%). The pdi values (<0.5) agrees with the result of morphological studies which showed that the particles were spherical, discrete and homogenously dispersed. The FTIR analysis verified that the SBs were properly encapsulated within the chitosan martrix. The XRD studies confirmed the nanoparticles as completely amorphous. The spectral results showed that there was an effective ionic complexation with strong interactions between the chitosan and the encapsulated biopeptides. The nanoparticles revealed sustained cumulative release for 12 h and improved efficacy with significantly higher (p < 0.05) ACE-inhibitory effect of the encapsulated compared to the unencapsulated biopeptides following simulated gastrointestinal digestion.
